# Leukemia-Associated Mutations in Nucleophosmin Alter Recognition by CRM1: Molecular Basis of Aberrant Transport

**DOI:** 10.1371/journal.pone.0130610

**Published:** 2015-06-19

**Authors:** Igor Arregi, Jorge Falces, Anne Olazabal-Herrero, Marián Alonso-Mariño, Stefka G. Taneva, José A. Rodríguez, María A. Urbaneja, Sonia Bañuelos

**Affiliations:** 1 Unidad de Biofísica (CSIC, UPV/EHU) and Department of Biochemistry and Molecular Biology, University of the Basque Country, Leioa, Spain; 2 Department of Genetics, Physical Anthropology and Animal Physiology, University of the Basque Country, Leioa, Spain; Universidad del Pais Vasco, SPAIN

## Abstract

Nucleophosmin (NPM) is a nucleocytoplasmic shuttling protein, normally enriched in nucleoli, that performs several activities related to cell growth. NPM mutations are characteristic of a subtype of acute myeloid leukemia (AML), where mutant NPM seems to play an oncogenic role. AML-associated NPM mutants exhibit altered subcellular traffic, being aberrantly located in the cytoplasm of leukoblasts. Exacerbated export of AML variants of NPM is mediated by the nuclear export receptor CRM1, and due, in part, to a mutationally acquired novel nuclear export signal (NES). To gain insight on the molecular basis of NPM transport in physiological and pathological conditions, we have evaluated the export efficiency of NPM in cells, and present new data indicating that, in normal conditions, wild type NPM is weakly exported by CRM1. On the other hand, we have found that AML-associated NPM mutants efficiently form complexes with CRM1^HA^ (a mutant CRM1 with higher affinity for NESs), and we have quantitatively analyzed CRM1^HA^ interaction with the NES motifs of these mutants, using fluorescence anisotropy and isothermal titration calorimetry. We have observed that the affinity of CRM1^HA^ for these NESs is similar, which may help to explain the transport properties of the mutants. We also describe NPM recognition by the import machinery. Our combined cellular and biophysical studies shed further light on the determinants of NPM traffic, and how it is dramatically altered by AML-related mutations.

## Introduction

**Fig 1:**
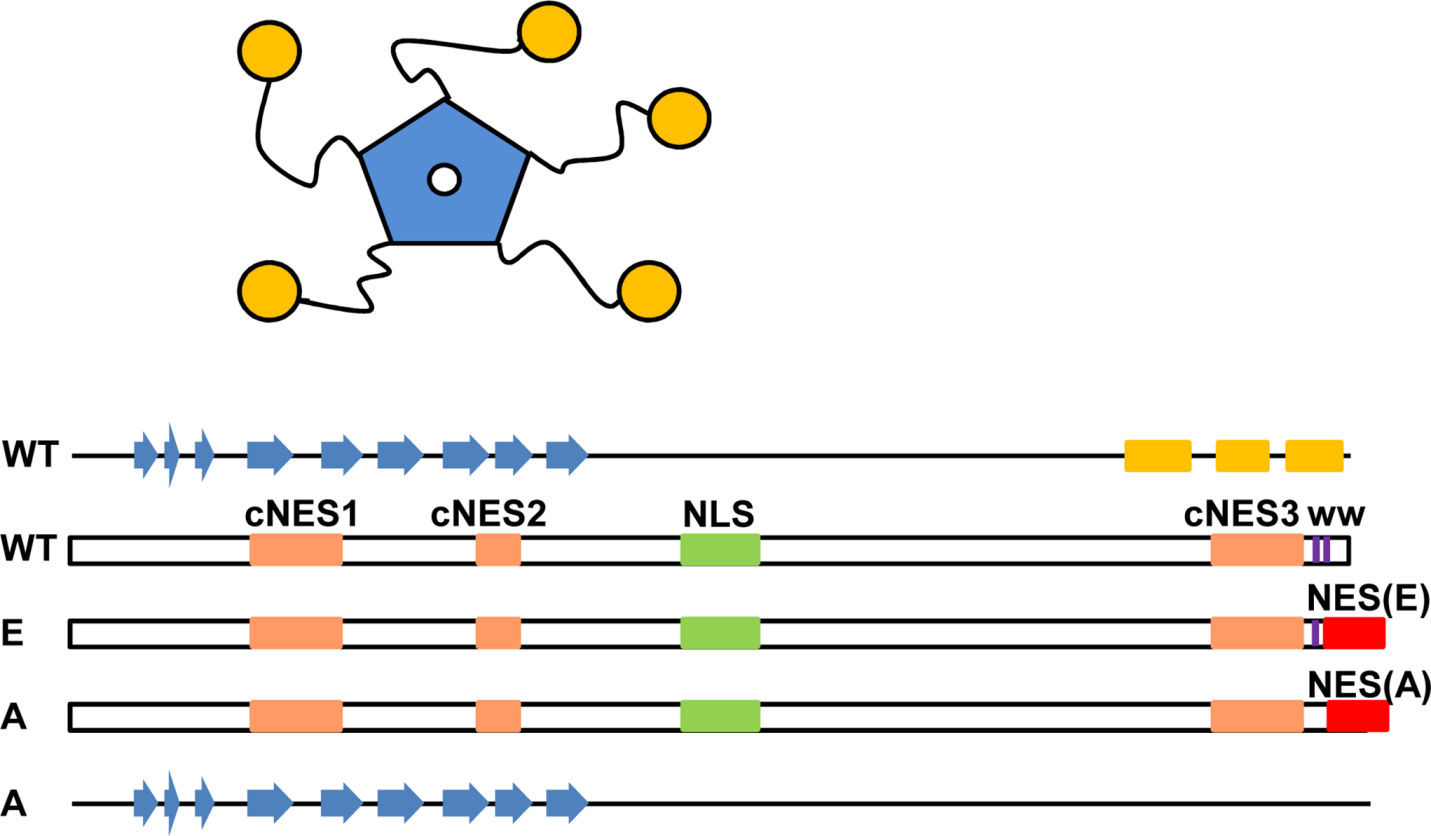
Schematic representation of NPM structure indicating the location of subcellular targeting motifs. **(A)** Schematic drawing of pentameric NPM, with core domain depicted in blue and C-terminal domains in yellow. **(B)** Position of candidate NES motifs (cNES) in NPM, in orange (cNES1 (42–61) [18], cNES2 (94–102) [3], and cNES3 (268–278) (this study)) and of acquired NES in AML-associated mutants A and E, in red. NLS (141–157) [3], in green, and the two Trp residues related to nucleolar targeting (288 and 290 [16], in purple) are also indicated. The secondary structure assignment of wild type NPM and mutant A are shown, β-strands denoted as blue arrows and α-helices as yellow cylinders. Mutant A is supposed to lack C-terminal helical structure, while mutant E would partly retain it [11].

Nucleophosmin (NPM, also termed NPM1, B23, NO38 or numatrin) is an abundant nucleolar protein responsible for several functions affecting cell growth [1]. NPM is involved in ribosome biogenesis and export of ribosomal subunits to the cytoplasm [2], controls centrosome duplication [3], regulates the stability of important tumor suppressors, such as p53 [4] and Arf [5], and participates in the cell response to stress, in particular, in DNA repair processes [6–8]. NPM is a pentameric, multidomain protein, composed of an N-terminal compact core of β-structure [9, 10] connected to small α-helical C-terminal domains [11] through long, flexible linkers (Fig 1). NPM interacts with a plethora of protein ligands [1], and indeed is considered a nucleolar hub [12]. It also binds nucleic acids [13], preferentially G-quadruplex structure-forming G-rich sequences [14].

Although normally enriched in nucleolus (specifically in the granular component of nucleoli that contains maturing preribosomal particles) [12], NPM constantly shuttles between cytoplasm, nucleoplasm and nucleolus [15]. Nucleolar retention of NPM has been related to the presence of two Trp residues near its C-terminal end [16], and could depend on binding of C-terminal domains to G-rich sequences in rDNA [17]. On the other hand, nucleocytoplasmic shuttling of NPM is signal and energy-dependent and is mediated by nuclear transport receptors. The linker region harbors a bipartite nuclear localization signal (NLS, residues 141–157) that can be recognized by importin α/β and mediates NPM nuclear import [3]. In addition, heterokaryon assays have shown that NPM nuclear export depends on the CRM1 (chromosome region maintenance 1) receptor [3, 18]. Less is known, however, about the molecular determinants of NPM export, e.g. the nuclear export signal(s) (NES) involved.

CRM1 is responsible for the nuclear export of many proteins bearing specific NESs [19]. CRM1 alone exhibits a very weak affinity for its cargos, which is significantly increased by the small GTPase Ran, in its GTP-bound form. Functional export complexes are, in fact, CRM1 / RanGTP / NES cargo ternary assemblies. A gradient of RanGTP (mostly bound to GTP inside the nucleus and to GDP in the cytoplasm) imparts directionality to both import and export events, controlling that the transport complexes assemble and disassemble in the proper compartment [20].

Classical NESs (so-called “Leu-rich”) consist of a stretch of 10–15 aminoacids with a specific pattern (Φ^1^-X_2-3_-Φ^2^-X_2-3_-Φ^3^-X-Φ^4^, where Φ stands for a hydrophobic residue) [19]. CRM1 is a toroidal protein built of helical HEAT repeats. Structural studies have elucidated how CRM1 recognizes and binds NESs through a hydrophobic, conserved groove formed between two α-helices on its convex surface [21–23]. These data have explained the previously described sequence requirements of an aminoacid motif to act as a NES, and have allowed re-definition of the NES consensus, including a further upstream hydrophobic residue, Φ^0^, that contributes to increase the binding strength [23].

Two sequence motifs in the core domain of NPM (residues 42–61 and 94–102) (Fig 1) have been investigated as candidate NESs [3, 18]. In addition, we have detected a further putative NES in the C-terminal region (see below). However, it is still unclear to what extent any of these sequences represents NPM physiological NESs. The amino acid sequence of these motifs does not strictly conform to the canonical NES consensus. More importantly, these motifs have either displayed border-line activity when tested in a well established nuclear export assay (residues 42–49 and 94–102) [24], or no binding to CRM1 (residues 42–61) [25]. Finally, these motifs, except for the most C-terminal one, are part of β-strands or extended regions of the compact core structure [9, 10] (Fig 1). Thus, their structural context seems unfavorable for direct binding to CRM1, unless a significant, not obvious, conformational change is invoked. It remains possible, nevertheless, that several of these proposed NES motifs may cooperate in the context of full length NPM to direct export, or may be potentiated by some regulatory mechanism such as phosphorylation [26]. Thus, further studies are required to explore the role of NPM putative NESs.

As mentioned above, the different activities of NPM are directly or indirectly related to cell growth. It is therefore not surprising that NPM deregulation is associated to cancer [6]. NPM is overexpressed in several solid tumors (such as gastric, colon, ovarian, prostate cancer). In addition, small insertions and translocations affecting *NPM1* gene, encoding NPM, are characteristic of certain hematological disorders [6]. In fact, *NPM1* is the most frequently mutated gene in adult acute myeloid leukemia (AML) [27, 28]. Approximately one third of AML patients display heterozygous *NPM1* gene mutations. In these patients, NPM is found aberrantly located in the cytoplasm of leukemic blasts (although part of NPM remains nucleolar, probably due to the formation of mixed wild type / mutant NPM oligomers). This aberrant cytoplasmic localization, which has been shown to be oncogenic [29, 30], is pathognomonic of an AML subtype [28]. AML-associated *NPM1* mutations are small insertions of a different number of bases at the end of the gene, causing a reading frameshift and generating a mutant NPM protein (“NPMc+”) with an altered aminoacid sequence. Depending on the mutation, the last 5–7 C-terminal residues ((WQ)WRKSL) are substituted for novel abnormal sequences of 9–11 aminoacids [27]. These mutations lead to the loss of at least one (as in the so-called mutant E) or more frequently both (as in mutant A) Trp residues. It has been shown that loss of these residues, crucial elements of the hydrophobic core of the C-terminal domain, impairs folding of the domain [11]. In turn, unfolding disrupts NPM nucleolar localization [11] due to inability to bind nucleic acids [17, 31]. These findings are consistent with the described requirement of those two Trp residues for the nucleolar localization of the wild type protein [16]. Additionally, the abnormal aminoacid sequence resulting from the mutation constitutes a novel NES that directs CRM1-mediated nuclear export of NPMc+. It has been clearly demonstrated that the acquired NES of NPMc+ is able to form a complex with CRM1 *in vitro* [25, 32], and that both factors (impaired nucleolar retention due to unfolding, and acquired NES activity) jointly contribute to aberrant cytoplasmic accumulation of mutant NPM in AML [28, 33]. Interestingly, the potency of the novel NESs acquired by different AML-associated NPM mutants, as determined using an *in vivo* nuclear export assay, seems to inversely correlate to the severity of their folding defect [24]. Thus, mutant A, which loses both Trp residues, has a relatively weak NES, while mutant E, which loses only one Trp and probably retains partial structure [11], has a more potent NES [24]. This observation further reinforces the view that the ability to be exported is critical for the oncogenic potential of mutant NPM. Importantly, whereas the potency of the NESs of AML mutants has been evaluated in cellular assays [24], the effect of these mutations on the NPM / CRM1 interaction strength remains to be directly established.

Here, we combine cellular and biochemical analyses to gain a deeper understanding of the molecular mechanisms involved in NPM subcellular localization. First, we have explored the export of wild type NPM in cellular assays, and observed that it is normally inefficient, but might be promoted by DNA damage. Next, we have evaluated the complex formation between CRM1^HA^ (a recently described [34] “high affinity” mutant of CRM1 that circumvents the technical difficulties posed by the weak affinity of wild type CRM1 for its ligands) and either wild type or mutant NPM by size exclusion chromatography (SEC). In addition, we have quantitatively assessed the CRM1^HA^ interaction with the NESs of different AML-associated mutants using both a fluorescence-based binding assay and isothermal titration calorimetry (ITC), in an attempt to rationalize their export behavior in terms of CRM1 binding properties. Finally, we have also thermodynamically characterized NPM binding to importin α/β in order to obtain a more complete view of the molecular basis of NPM nucleocytoplasmic transport.

## Materials and Methods

### Plasmids and cloning procedures

The clone of Myc3 tagged human NPM in pCDNA3.1 for eukaryotic expression was kindly provided by Dr. Yanping Zhang (University of Texas) [35]. From this clone, we used PCR to generate the AML-associated mutants A and E [27], by incorporating the mutations in the 3´oligonucleotide. The three NPM variants (wild type, A and E) were subcloned as YFP-NPM fusions into pEYFP-C1. The plasmid encoding YFP-CRM1 has been previously described [36], and pEYFP-C1 was used as its corresponding negative control. As a positive control in cell localization assays, a previously described plasmid encoding Flag-tagged Survivin fused to a heterologous NLS (“SurvNLS”) [37] was used. To test the export activity of a novel putative NES in the C-terminal domain of wild type NPM, a DNA fragment encoding residues 264–282 was cloned into the plasmid Rev (1.4)-GFP (a gift of Dr. Beric Henderson, University of Sydney), as previously described [38].

For recombinant production in *E*. *coli*, we subcloned wild type NPM, as well as mutants A and E, as N-terminal (His)_10_ –ZZ tagged constructs in pTG-A20 vector [22]. The original clone of wild type mouse CRM1, N-terminally fused to a His—ZZ tag [22] was a kind gift of Dr. Dirk Görlich (Max Planck Institute for Biophysical Chemistry, Göttingen). We generated the high affinity CRM1 mutant (CRM1^HA^) [34] from a clone provided by Dr. Murray Stewart (MRC Laboratory of Molecular Biology, Cambridge). For generation of His tagged TFP-NES constructs, double-stranded DNA fragments encoding the NES motifs LALKLAGLDI (TFP-PKI(NES)), DLCLAVEEVSLRK (TFP-mutA(NES)) and DLWQSLAQVSLRK (TFP-mutE(NES)) preceded by the spacer ISGGGG were generated by annealing overlapping oligonucleotides, followed by extension with Klenow DNA polymerase and digestion with BglII and XhoI. Then, the fragments were inserted into the BglII / XhoI sites of a plasmid derived from pPROEX-HTc (Life Technologies) and coding for mTFP1, a modified, monomeric version of teal fluorescent protein (a cyan fluorescent protein), provided by Dr. Álvaro Villarroel (Unidad de Biofísica, Bilbao). The C-terminal sequence of wild type NPM, DLWQWRKSL, was also cloned in this plasmid to be used as a negative control. For direct comparison of binding parameters with published data, the previously described YFP-PKI(NES) [39], kindly provided by Dr. Yoshiyuki Matsuura (Nagoya University) was used. All clones were checked by DNA sequencing.

### Cell culture, transfection, treatment and imaging

Human embryonic kidney 293T (HEK293T) and HeLa cells (ECACC) were grown in Dulbecco’s modified Eagle’s medium, supplemented with 10% fetal bovine serum, 100 units/ml penicillin and 100 μg/ml streptomycin (all from Invitrogen). The cells were seeded onto sterile glass coverslips in 12-well trays 24 h before transfection. Transfections were carried out with XtremeGENE 9 (Roche) following the manufacturer’s protocol. Treatment of cells with the different drugs was done at the indicated final concentrations and time periods: Actinomycin D (ActD, Sigma) at 5 μg / mL for 3 h, and hydroxyurea (HU, Sigma) at 4 mM for 24 h.

Cells were fixed, either 20 or 42 h after transfection, with 3.7% formaldehyde in PBS for 30 min, then treated with 0.2% Triton X-100 in PBS for 10 min, for membrane permeabilization and incubated for 1 h in blocking solution (3% BSA in PBS). In YFP-CRM1 overexpression experiments, endogenous NPM was detected with anti-B23 (Santa Cruz Biotechnology FC82291, dilution 1:800), Myc3-NPM with anti-Myc (Cell Signalling 2276, dilution 1:2000) and Flag-SurvNLS with anti-Flag (Sigma F1704, dilution 1:300). Primary antibodies were diluted in blocking solution. In all cases, the secondary antibody was Alexa Fluor 594 anti-mouse (Invitrogen A11005, dilution 1:400). Washing between the different steps were carried out with PBS. Cells were finally mounted on to microscope slides using Vectashield containing DAPI (Vector). Slides were examined using a Zeiss Axioskop fluorescence microscope. For quantification, at least 200 cells were “blind counted” and classified as having nuclear (or nucleolar), nuclear/cytoplasmic or cytoplasmic localization of NPM (or the Flag-SurvNLS control). Images were taken with an Olympus Fluoview FV500 confocal microscope equipped with FV-viewer software.

The export activity of a novel putative NES in the C-terminal domain of NPM was tested using an established *in vivo* nuclear export assay in HeLa cells [38] (see Supporting Information for further experimental details).

### Protein production

Expression of all proteins in *Escherichia coli* (BL21 (DE3) strain for NPM and TFP-NES contructs, and BL21-CodonPlus (DE3) strain for CRM1), was induced with 1 mM IPTG and carried out overnight at 18°C. For purification, cells were disrupted by sonication in the following buffers, supplemented in all cases with lysozyme (20 mg/L culture) and protease inhibitors (cOmplete ULTRA, Roche, for NPM and cOmplete, EDTA-free, Roche, for CRM1 and TFP/YFP-NES constructs): 25 mM Tris/HCl, pH 7.5, 500 mM NaCl, 20 mM imidazole, 5 mM MgCl_2_, 1 mM TCEP, 10% glycerol (NPM and mutants thereof), 50 mM Tris/HCl, pH 8.0, 500 mM NaCl, 15 mM imidazole, 1 mM MgCl_2_, 1 mM TCEP, 5% glycerol (CRM1) and 50 mM potassium phosphate, pH 7.0, 200 mM NaCl, 20 mM imidazole, 1 mM MgCl_2_, 2 mM TCEP (TFP/YFP-NES). The clarified extract was loaded on a Ni—NTA affinity column (cOmplete His-Tag Purification Column, Roche, for NPM and mutants, and Histrap FF, GE Healthcare for CRM1 and TFP/YFP-NES) and the protein eluted with an imidazole gradient. The His-ZZ tag was removed from CRM1 and NPM through proteolysis with TEV, and the untagged product isolated with a second Ni-NTA chromatography. The proteins were then further purified by gel filtration chromatography with Superdex 200 (GE Healthcare) in 25 mM Tris/HCl, pH 7.5, 500 mM NaCl, 1 mM DTT, 10% glycerol (in the case of NPM), 20 mM Tris/HCl, pH 7.4, 50 mM NaCl, 5 mM DTT, 2 mM MgCl_2_ (CRM1), and 50 mM Tris/HCl, pH 7.4, 100 mM NaCl, 2 mM DTT, 2 mM MgCl_2_ (TFP/YFP-NES constructs). Finally they were concentrated and flash-frozen for storage. Protein concentration was determined with the BCA colorimetric assay (Pierce) and spectrophotometrically. The integrity of the produced proteins was checked by mass spectrometry, and partial proteolytic degradation was observed in the case of full length mutants A and E (see below and in Supporting Information). Importins (α/β and Δα, lacking the importin β binding (IBB) motif), were obtained with a very similar protocol [40].

### Circular Dichroism (CD)

CD spectra were recorded with a Jasco 720 circular dichroism spectropolarimeter, using quartz cuvettes of 1 mm path length, in buffer 20 mM Tris/HCl, pH 7.4, 50 mM NaCl, 5 mM DTT, 2 mM MgCl_2_. Protein concentration was 2.5 μM. Temperature scans were done at 60°C /h. Mean residue ellipticity values were calculated using the expression Θ = ε/10*cln*, where ε is the ellipticity (millidegrees), *c* the protein concentration (mol/L), *l* is the path length (cm), and *n* is the number of peptide bonds of the protein.

### Chromatography-based binding experiments

For size exclusion chromatography, NPM (either wild type or mutants A and E) at 20 μM (pentamer), was mixed with 40 μM CRM1^HA^, incubated for 30 min at room temperature, and centrifuged 15 minutes at 14500 r.p.m. Then 100 μL were loaded onto a Superdex 200 10/30 column (GE Healthcare), equilibrated in buffer 20 mM Tris/HCl, pH 7.4, 100 mM NaCl, 5 mM DTT, 2 mM MgCl_2_. Runs were performed at 15°C and a flow rate of 0.2 mL / min. The eluted fractions were analyzed by SDS-PAGE. TFP-NES/CRM1^HA^ complex formation was also tested by Ni-NTA chromatography (see Supporting Information).

### Fluorescence anisotropy binding assays

To estimate CRM1 binding affinities for NES motifs we monitored binding with a fluorescence-polarization assay based on previously described approaches [23, 34, 41]. The increase in fluorescence anisotropy of TFP/YFP when the TFP/YFP-NES construct (50 nM) binds to 0–60 μM CRM1 (either CRM^HA^ or wild type) was recorded at 25°C with a 8100 SLM-Aminco spectrofluorometer in buffer 20 mM Tris/HCl, pH 7.4, 50 mM NaCl, 5 mM DTT, 2 mM MgCl_2_. Excitation and emission wavelengths were 463, 500 nm for TFP and 463, 533 nm for YFP, and slits width 4 and 8 nm, respectively.

### Isothermal titration calorimetry (ITC)

ITC measurements were carried out using a VIP-ITC MicroCalorimeter (MicroCal, Inc., Northampton, MA). TFP and YFP constructs, as well as CRM1^HA^, were dialyzed in a buffer containing 20 mM HEPES, pH 7.4, 50 mM NaCl, 1 mM TCEP, 1 mM MgCl_2_, and degassed prior to the experiment. Untagged peptides (AS: QDLCLAVEEVSLRK, AL: EAIQDLCLAVEEVSLRK, ES: QDLWQSLAQVSLRK and EL: EAIQDLWQSLAQVSLRK), N-acetylated and 95% pure, were purchased from Proteogenix (Schiltigheim, France) and resuspended in the ITC buffer. Measurements were performed at 20°C. TFP-NES, YFP-NES or NES peptides at 35–180 μM were titrated onto CRM1^HA^ at 2–14 μM. Injections of 6 μL were performed at 320 seconds interval (the first injection always was of 1 μL and is not considered in data analysis). To determine the thermodynamic parameters of NPM / importin interaction, importin α, importin Δα or importin α/β at 45–60 μM were titrated onto NPM (1–1.2 μM pentamer) in 50 mM Tris/HCl, pH 7.5, 100 mM NaCl, 2 mM TCEP, and 10% glycerol. The importin α/β heterodimer was prepared with an excess of importin β, which does not bind to NPM, to avoid the presence of free importin α. “Reverse” titrations, where 20–30 μM NPM was titrated onto 7–10 μM of the different importins were also performed and yielded similar results.

The reaction heat of each injection is related to the calorimetric enthalpy of binding, ΔH^0^. The binding isotherms, ΔH^0^ *vs*. molar ratio, were analyzed with an independent binding sites model using MicroCal Origin software. The fit of the binding curve yields the binding constant K_A_ (K_D_ = 1/K_A_) and the enthalpy ΔH^0^ of the binding reaction. The Gibbs free energy of binding ΔG^0^ and the entropy ΔS^0^ are determined from the basic thermodynamic expressions: ΔG^0^ = -RTlnK_A_ = ΔH^0^−TΔS^0^, where R and T are the gas constant and the absolute temperature, respectively.

## Results

### NPM is inefficiently exported to the cytoplasm by CRM1

The predominantly nucleolar localization of NPM is likely due to a combination of nucleolar retention and inefficient CRM1-mediated export. In order to gauge the relative contribution of both factors, we examined the subcellular localization of endogenous and ectopically expressed NPM in conditions where its nucleolar retention is impaired by treatment with actinomycin D (ActD), a drug that disrupts nucleoli, and the CRM1-mediated export is promoted by CRM1 overexpression.

**Fig 2:**
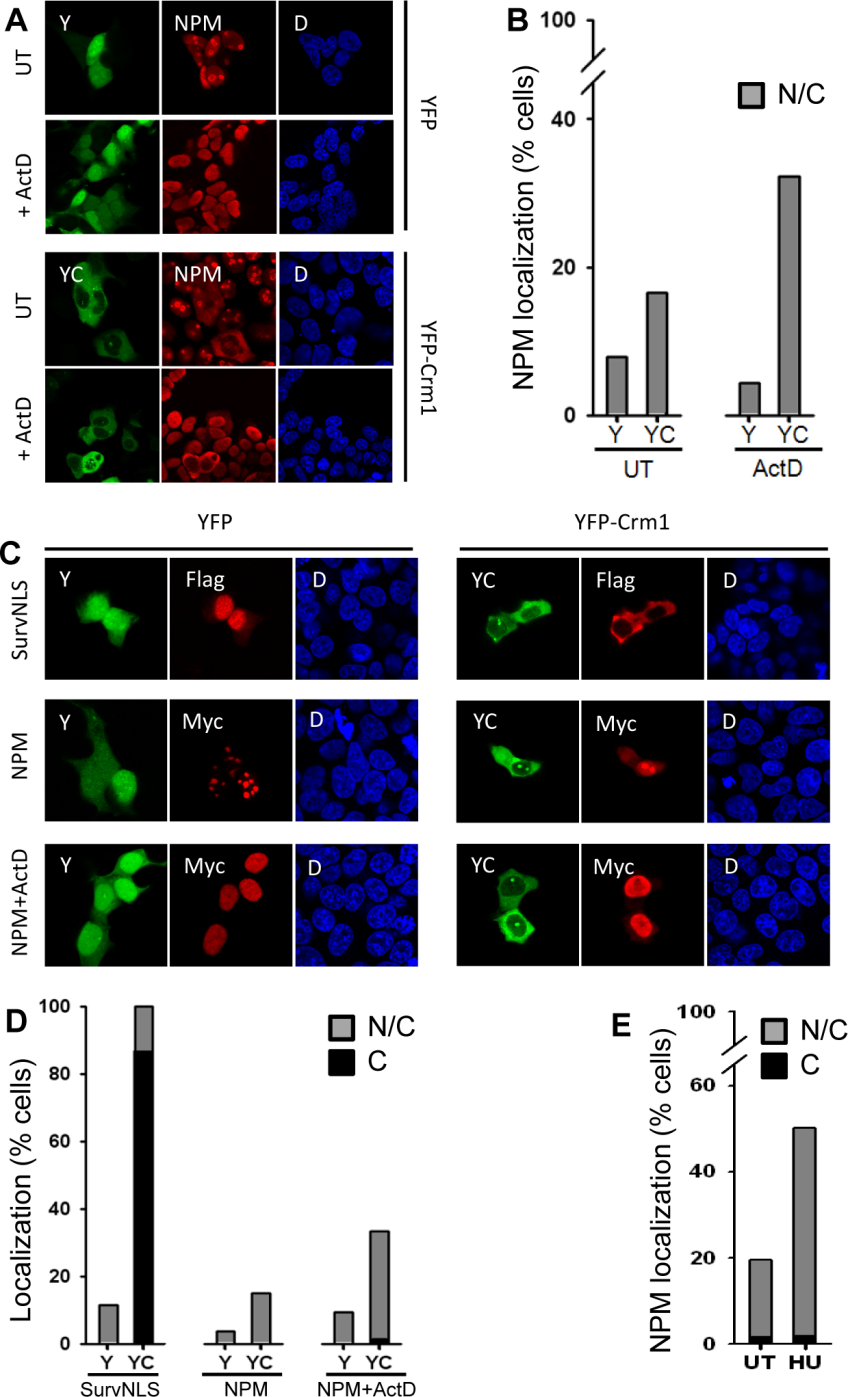
Localization of endogenous and ectopically expressed NPM and effect of treatment with actinomycin D (ActD) and CRM1 overexpression in HEK293T cells. **(A)** Localization of endogenous NPM in cells overexpressing YFP-CRM1 (or YFP alone) and either untreated (UT) or treated with ActD. Green denotes YFP (Y) or YFP-CRM1 (YC) fluorescence; red, immunostaining of endogenous NPM; and blue, DAPI fluorescence. **(B)** Percentage of cells showing nuclear and cytoplasmic (N/C) localization of NPM. More than 200 cells were “blind” counted. **(C)** Localization of Flag-SurvNLS and Myc-NPM when co-expressed with YFP alone (control) or YFP-CRM1, and effect of ActD. **(D)** Quantification of cytoplasmic (C) or nuclear and cytoplasmic (N/C) localization of overexpressed Flag-SurvNLS and Myc-NPM. **(E)** Effect of treatment with hydroxyurea during 24 h. on the localization of Myc-NPM in cells expressing YFP-CRM1.

In untreated HEK293T cells expressing the empty YFP vector (negative control), endogenous NPM is mostly located in the nucleoli (Fig 2A), as expected, although a faint nucleoplasmic signal can also be detected. Furthermore, consistent with the described enrichment of NPM in the granular compartment of nucleolus [12], a more intense NPM immunostaining is detected in the nucleolar periphery. A semiquantitative assessment revealed that only 8% of the cells (excluding apoptotic or damaged cells and cells undergoing mitosis) show partial cytoplasmic localization of endogenous NPM (Fig 2B). ActD is known to block transcription and disrupt nucleolar structure, causing the spread of several nucleolar components through the nucleoplasm, and it has been shown to favor nuclear export of some nucleolar proteins [38]. We hypothesized that ActD-mediated release of NPM from nucleoli might enhance availability for CRM1 binding in the nucleoplasm, and therefore facilitate its export. In YFP-transfected, ActD-treated cells, NPM staining is indeed observed through the entire nuclei (Fig 2A). This does not affect, however, the amount of cells displaying nuclear/cytoplasmic localization of NPM (Fig 2B). These results indicate that, in these conditions, endogenous NPM export in HEK293T cells is inefficient, even when its nucleolar retention is abrogated.

It has been shown that nucleocytoplasmic shuttling proteins that are predominantly located in the nucleus may translocate to the cytoplasm upon overexpression of CRM1 [36]. Indeed, ectopically expressed YFP-CRM1 increases cytoplasmic localization of endogenous NPM, and a further increase was observed upon ActD treatment (Fig 2A and 2B). These observations are in agreement with the described CRM1-mediated shuttling of NPM [3, 18], and suggest that, among other factors, both availability of NPM in the nucleoplasm, and CRM1 protein levels may limit export of the protein. It is important to note that NPM export is nevertheless inefficient, since even in the presence of ectopically expressed CRM1 and after treatment with ActD, endogenous NPM remains predominantly nuclear in most cells.

Similar results were obtained when the localization of Myc3-tagged NPM was analyzed under the same experimental conditions. Thus, the localization of Myc-NPM was almost exclusively nucleolar in untreated cells expressing YFP, but co-expression with YFP-CRM1 and treatment with ActD resulted in a higher proportion of cells with nuclear/cytoplasmic Myc-NPM (Fig 2C and 2D). In these experiments, a Flag-SurvNLS chimeric protein was also tested as a reference for the effect of YFP-CRM1 co-expression on the localization of Myc-NPM. Flag-SurvNLS is a version of the nucleocytoplasmic shuttling protein Survivin that bears a potent heterologous NLS and is predominantly located in the nucleus, but readily relocates to the cytoplasm when co-expressed with YFP-CRM1 [37]. In comparison to nearly complete cytoplasmic relocation of Flag-SurvNLS, only 33% of the cells exhibited nuclear/cytoplasmic localization of Myc-NPM in the most “export-prone” conditions (YFP-CRM1 co-expression and treatment with ActD) (Fig 2C and 2D).

Altogether, the results above show that CRM1-mediated NPM export is inefficient. In fact, the aminoacid sequence determinants responsible for NPM export are still unclear. Using a new NES-prediction tool, termed Wregex [42], we identified a candidate NES motif (268-FINYVKNCFRM-278) in the C-terminal domain of NPM (Fig 1) that has not been previously investigated. However, this predicted NES was found to be inactive when tested in an *in vivo* nuclear export assay [38] (S1 Fig), and it is therefore unlikely to contribute to NPM export.

Finally, given the role of NPM in DNA repair [6–8], we tested the possibility that DNA damage, which has been described to promote release of NPM from nucleoli [7], might favor its nuclear export. As shown in Fig 2E, the cytoplasmic relocation of Myc-NPM induced by YFP-CRM1 co-expression was more pronounced (increasing from 20% to 50%) when cells were treated with hydroxyurea (HU), a drug that induces DNA lesions. Interestingly, HU appears to facilitate NPM export more efficiently than ActD, indicating that other mechanisms, apart from nucleolar release of NPM, might be involved. However, the effect of HU could not be observed on endogenous NPM (data not shown), and therefore the putative modulation of NPM localization by DNA damage remains to be further established.

### AML-associated NPM mutants form stable complexes with CRM1^HA^

**Fig 3:**
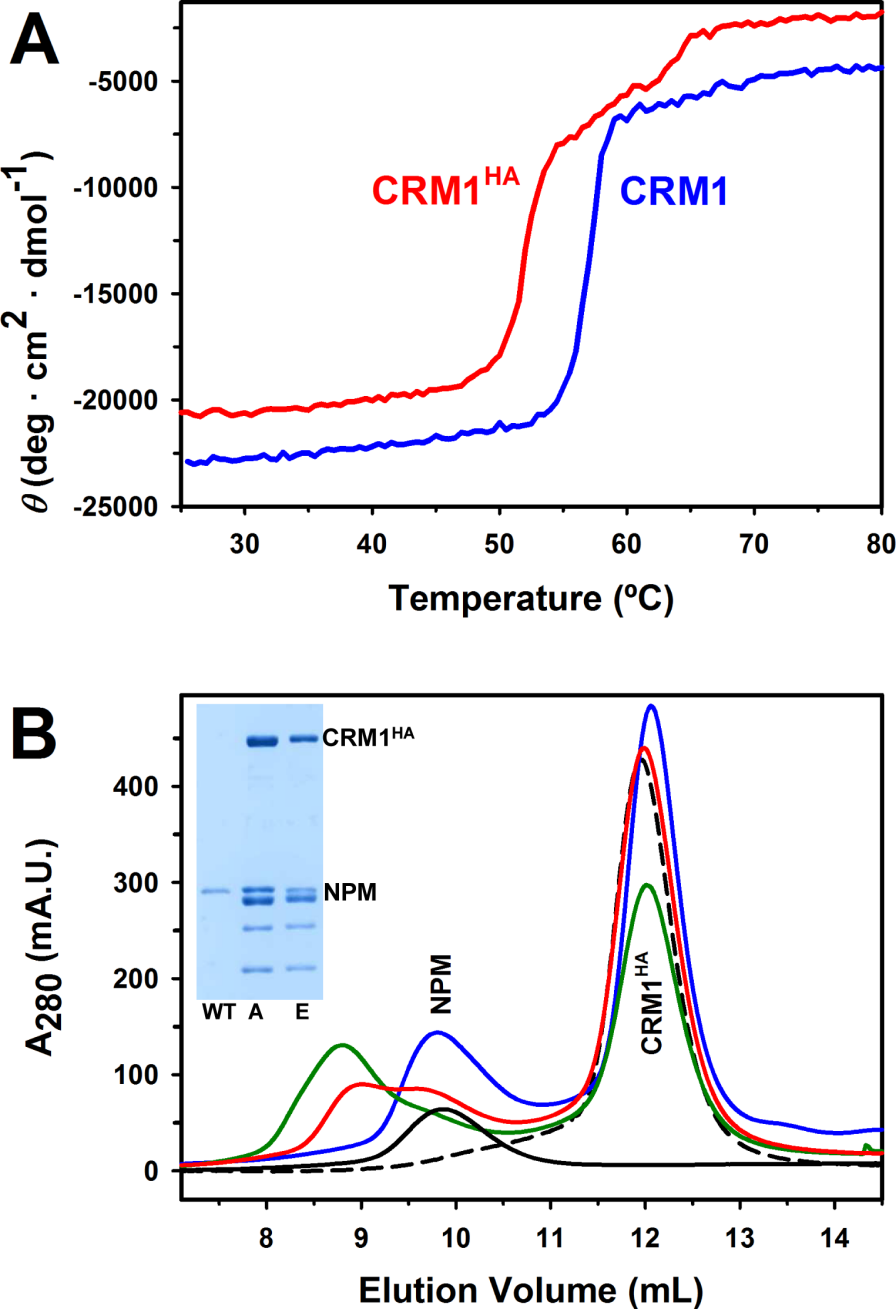
Native-like properties of CRM1^HA^ “high affinity” mutant [34] and formation of complexes between this CRM1 variant and AML-related NPM mutants. **(A)** Circular dichroism thermal denaturation profiles of wild type CRM1 and CRM1^HA^, followed by ellipticity at 222 nm as a function of temperature. **(B)** Elution profiles of mixtures of 40 μM CRM1^HA^ with 20 μM (pentamer) of wild type NPM (blue), NPMmutA (green) and NPMmutE (red), as well as of the proteins alone (black) (the elution behavior of the three NPM variants is very similar, and only NPMmutE is shown for the sake of clarity). Samples were run in a Superdex 200 HR 10/30 column. When evaluating the relative amounts of the complex and free NPM, one should take into account that absorbance at 280 nm is lower for the mutants (especially NPMmutA) than for wild type NPM, since wild type NPM contains two Trp residues per subunit, NPMmutE one, and NPMmutA none. Inset: SDS-PAGE of fractions of the three mixtures eluting between 8.5 and 9 mL. The samples of NPMmutA and NPMmutE contain a high proportion of proteolytic products, most probably C-terminally truncated and therefore lacking the NES (see text and S3 Fig). They are detected in the fractions containing the complex probably due to formation of mixed oligomers with the full length species and/or incomplete resolution from free NPM.

We next attempted to characterize the *in vitro* complex formation between NPM and CRM1. Considering the low intrinsic affinity of CRM1 for its cargos [20], we decided to use a CRM1 mutant that has been described to bind NES cargos with 600 times higher affinity than wild type CRM1 [34], independently of RanGTP. This “high affinity” mutant, which we term “CRM1^HA^”, lacks the nine last C-terminal residues and has three hydrophobic residues (^430^VLV^432^) substituted for alanine, both regions being crucial in the regulatory cycle of the protein [34]. The use of this mutant is a convenient approach to the *in vitro* characterization of CRM1 complexes, since significant NES binding can be detected even in the absence of the otherwise required RanGTP [34]. We have confirmed, by circular dichroism, that the “HA” mutations do not significantly modify CRM1 secondary structure (not shown), but slightly compromise its thermal stability (Fig 3A). This result is consistent with the slight destabilization of a C-terminally truncated CRM1 reported elsewhere [41].

We first tried to detect binding of wild type NPM to CRM1^HA^ by size exclusion chromatography (SEC), an approach we have previously used to study the nucleoplasmin / importin interaction [40]. A mixture of 20 μM (pentamer) NPM and 40 μM CRM1^HA^ did not result in any complex stable enough as to be detected by SEC (Fig 3B). Remarkably, a mixture of 40 μM NPM and 140 μM CRM1^HA^ also failed to produce any detectable complex (data not shown). This observation suggests a very low or null affinity of wild type NPM for the export receptor.

Several NPM mutants associated with AML have been identified [27]. We have focused our study on two of these mutants, one termed A (NPMmutA), which is the most frequently detected in NPMc+ AML, and the other termed E (NPMmutE). In agreement with published reports [33], we have observed that YFP-NPMmutA is more cytoplasmic in transfected HeLa cells than YFP-NPMmutE, which remains partly nucleolar (S2 Fig). Based on the results from *in vivo* export assays, it has been reported that a more potent acquired NES in NPMmutE counteracts the higher affinity for nucleoli of this mutant with respect to NPMmutA, and ensures its efficient cytoplasmic relocation [24]. However, while the relative potency of the isolated NESs has been compared in cellular assays, the ability of the full length mutant NPM proteins to bind CRM1 has not yet been tested. In order to explore the CRM1 binding properties of AML-related mutants, we produced recombinant NPMmutA and NPMmutE. As one might predict given their folding defect [11], we were not able to completely avoid proteolytic degradation: Our preparations of recombinant NPM mutants include a high proportion of C-terminally degraded species, as determined by mass spectrometry analysis (S3 Fig). In spite of this limitation, and in contrast to wild type NPM, complexes of NPMmutA and NPMmutE with CRM1^HA^ can be readily detected by chromatography (Fig 3B). Although not saturated, and not fully resolved from unbound NPM, the formation of these complexes is evident from the population of larger size (i.e., eluting earlier from the column) containing both CRM1^HA^ and mutant NPM, as checked by SDS-PAGE (Fig 3B, inset). Surprisingly, considering the described higher export activity of NPMmutE NES motif [24], both the chromatographic profiles and the relative intensity of the electrophoretic bands suggest that the CRM1^HA^ / NPMmutA complex forms with higher yield than CRM1^HA^ / NPMmutE. Unfortunately, the aforementioned proteolytic degradation of the mutants, also evident in the shown gel (Fig 3B, inset), precludes further biophysical analysis, such as estimating the binding stoichiometry (i.e. how many CRM1^HA^ molecules can bind to the NPM oligomer) and complicates the interpretation of the results. In this regard, one should bear in mind that the truncated species are not expected to bind CRM1^HA^ better than wild type NPM, but are present in these fractions probably due to formation of mixed oligomers with the full length species, and/or to incomplete resolution of the complex from the population of free NPM. Of note, NPMmutE seems to be more degraded than NPMmutA (Fig 3B, inset, and S3 Fig), which may contribute to its apparently weaker binding. In line with this view, comparison of the binding of two separate NPMmutA preparations exhibiting different degree of proteolytic degradation indicates that the amount of complex formed correlates with the relative concentration of the full length species (not shown). On the other hand, the fact that mutant E is expected to partly keep the structure of the C-terminal domain [11] might also explain its poorer binding to CRM1^HA^ due to a less accessible NES. It is remarkable that even the limited amount of full length molecules of NPM mutants present in the samples (effective concentration much lower than nominal) is enough to form stable complexes with CRM1^HA^ readily detected by SEC. We conclude that the AML-associated NPM mutants A and E display significantly higher affinity for CRM1^HA^ than wild type NPM, although the technical problems encountered do not allow us to ascertain if any mutant (A or E) is better recognized by CRM1^HA^.

### The acquired NES motifs of AML-associated NPM mutants display different affinities for CRM1

**Fig 4:**
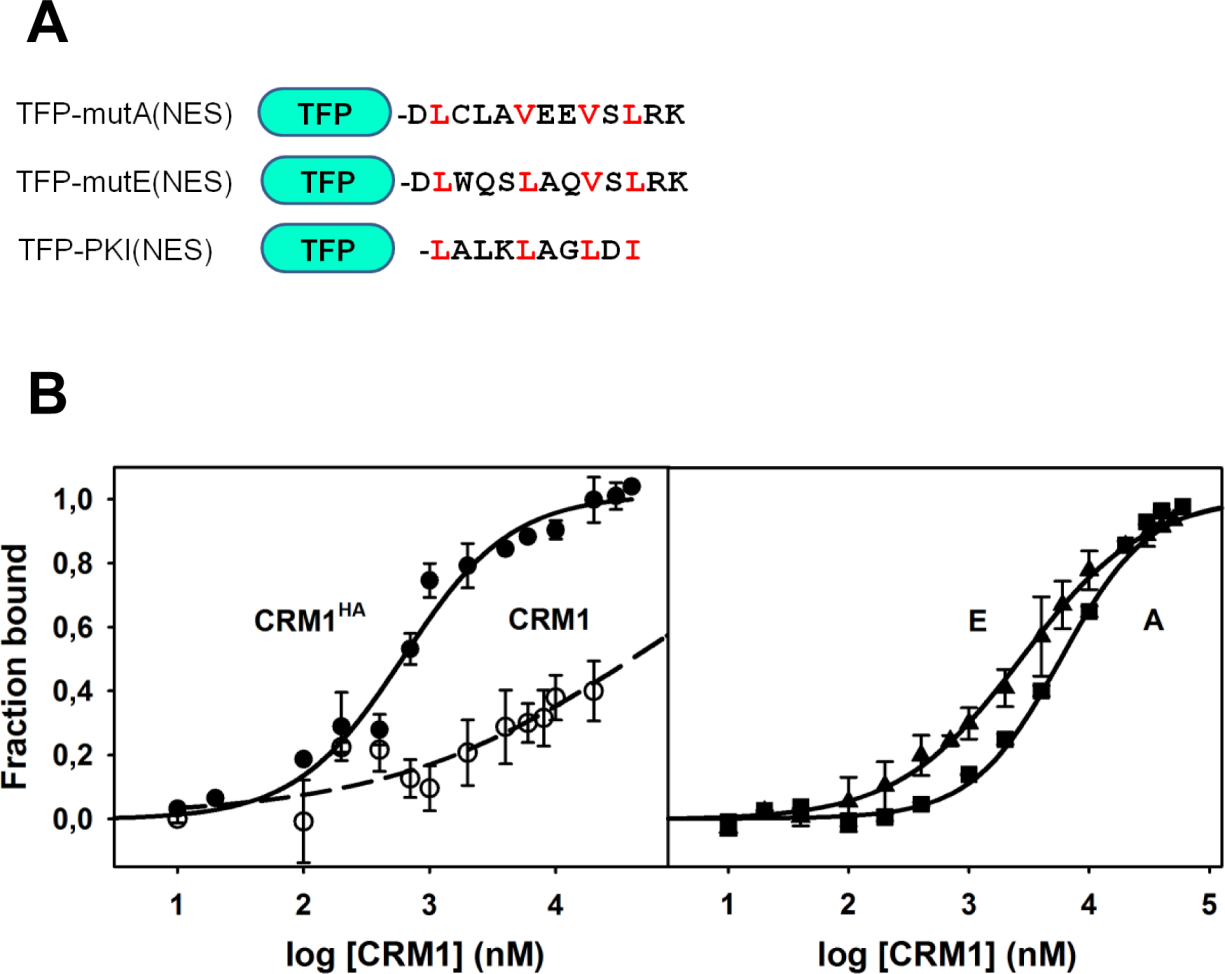
Binding of CRM1 to TFP-NES constructs. **(A)** Scheme of the engineered TFP constructs, showing the sequence of the NES motifs. Φ residues are highlighted in red. **(B)** Titration of CRM1 onto the different TFP constructs (50 nM), at 25°C, in buffer 20 mM Tris/HCl, pH 7.4, 50 mM NaCl, 5 mM DTT, 2 mM MgCl_2_: Left panel: Binding of CRM1^HA^ (solid symbols) or wild type CRM1 (empty symbols) to TFP-PKI(NES). Right panel: Binding of CRM1^HA^ to TFP-mutE(NES) (triangles) and TFP-mutA(NES) (squares). Fraction bound was calculated from the relative increase in fluorescence anisotropy of TFP (λ_exc_ 463 nm, λ_em_ 500 nm). Error bars correspond to at least three measurements per CRM1 concentration.

Although the relative potency of the NESs acquired by the different NPM mutants has been compared using an *in vivo* export assay [24], their CRM1-binding affinities have not yet been determined. We cloned the NESs of mutant A and E as C-terminal fusions to a teal fluorescent protein (TFP) (Fig 4A). As a reference, we generated an equivalent construct with the well-characterized NES of protein kinase inhibitor (PKI) [23]. We first tested the binding of CRM1^HA^ to the TFP-NES constructs using Ni-NTA chromatography. His-tagged TFP constructs were bound to the Ni-NTA column, and an excess of untagged CRM1^HA^ was then injected (S4 Fig). Comparison of the amounts of bound (i.e. coeluting with TFP-NES) and unbound CRM1^HA^ indicates that CRM1^HA^ binding to TFP-PKI(NES) is stronger than its binding to TFP- mutE(NES), whereas binding to TFP- mutA(NES) is not even detected in these conditions. This result suggests a ranking of affinities for CRM1^HA^: PKI(NES) > mutE(NES) > mutA(NES). Therefore, in the context of the TFP-(NES) constructs, CRM1^HA^ displays higher avidity for the NES of mutant E.

We next used the TFP-(NES) constructs to monitor CRM1^HA^ binding to each motif, using the change in fluorescence anisotropy upon complex formation as a readout (Fig 4B), as previously described [23, 34, 41]. For comparison, the binding of wild type CRM1 to TFP-PKI, much weaker than that of CRM1^HA^, as expected [34], is also shown. Of note, the affinity we estimate for CRM1^HA^ binding to TFP-PKI(NES) (K_D_ 579 nM) is lower than previously described (K_D_ 25 nM) for a similar fluorescent construct of YFP-PKI(NES) [34]. The different nature of the fluorescent tag, and/or the linker between the fluorescent protein and the NES (2 residues longer in the YFP-PKI construct) could explain the discrepancy. In fact, when using the same YFP-PKI(NES) construct tested by Fox *et al*. [34, 39], we have observed a higher affinity by fluorescence anisotropy (K_D_ 112 nM) and by ITC (K_D_ 18 nM, see below). These observations highlight the relevance of the molecular context in which the CRM1-NES interaction is studied, suggesting that accurate comparison of relative affinities between different NES motifs would require that they are assayed in the same context. To isolate the CRM1 / NES interaction under study from the influence of the tag protein, we have also conducted binding assays with peptides corresponding to the NES motifs (see below).

In the fluorescence anisotropy assay, CRM1^HA^ was found to bind TFP-mutE(NES) with slightly higher affinity than TFP-mutA(NES) (apparent K_D_ of 2.2 μM *vs*. 5.0 μM, Fig 4B and Table 1). The higher affinity of mutE(NES) for CRM1^HA^ is consistent with its stronger export activity previously observed using *in vivo* nuclear export assays [24]. However, in these previously reported assays the export activity of mutant E NES was found to be much higher than that of mutant A NES, and comparable to the export activity of PKI(NES) [24]. Thus, the ranking of *in vitro* CRM1^HA^ affinity (PKI(NES) (K_D_ 579 nM) > mutE(NES) (2.2 μM) > mutA(NES) (5.0 μM)) is only partially in line with the ranking of NES potency determined in cellular export assays (PKI(NES) = mutE(NES) >> mutA(NES)) [24]. These findings suggest that the results of cell-based nuclear export assays do not necessarily correlate with CRM1 binding affinity parameters measured *in vitro*.

**Table 1:** Affinities (apparent K_D_) of the different complexes studied, as estimated by fluorescence anisotropy titrations.

TFP/YFP construct	K_D_ (nM)^(1)^
**YFP-PKI(NES)**	112 ± 87
**TFP-PKI(NES)**	579 ± 56
**TFP-mutE(NES)**	2162 ± 556
**TFP-mutA(NES)**	4966 ± 1181

^(1)^ Apparent K_D_ (C_50_).

### Thermodynamic characterization of CRM1/NES interactions

**Fig 5:**
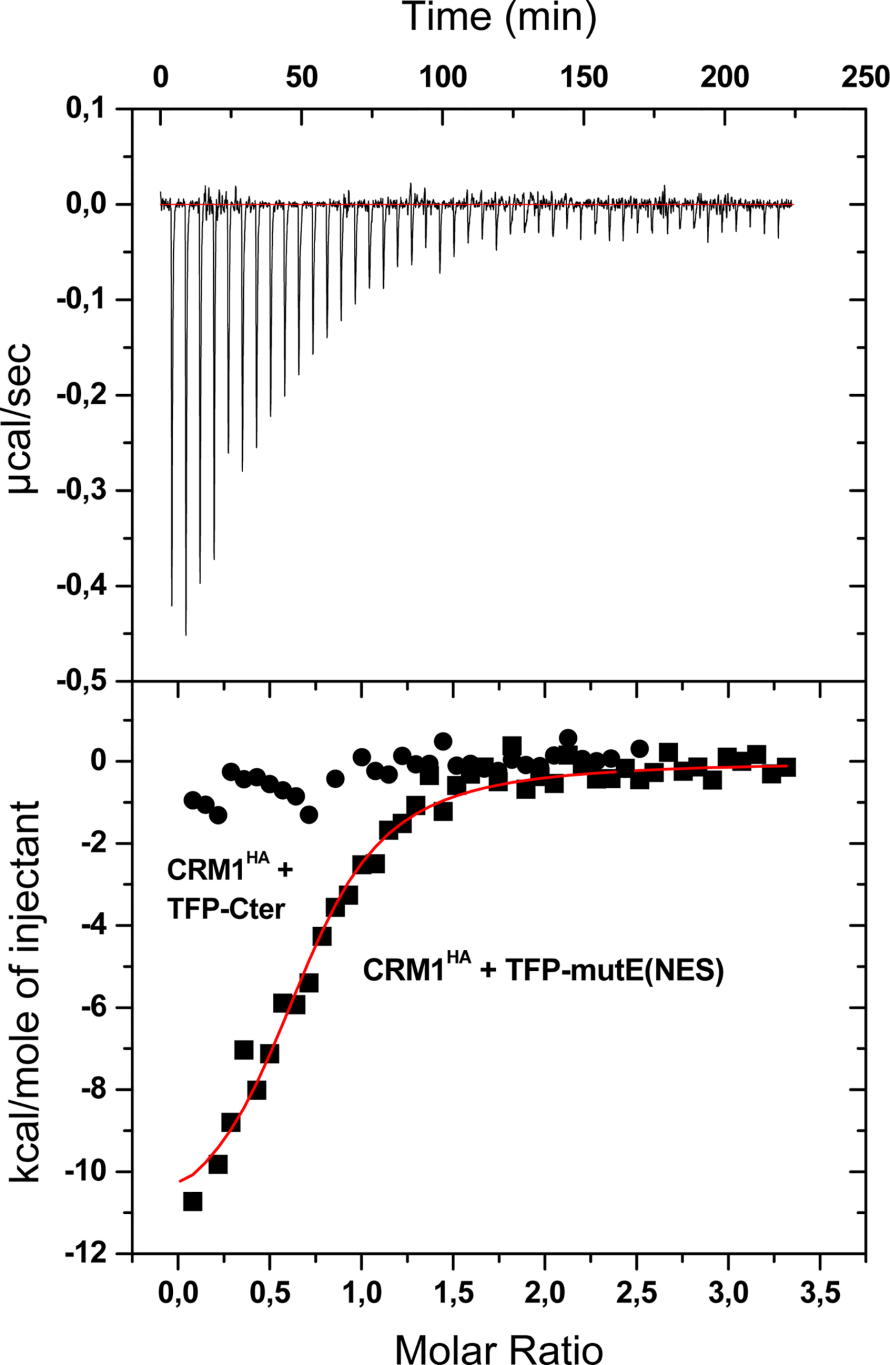
Analysis of CRM1^HA^/TFP-NES interaction by ITC. Isothermal titration of CRM1^HA^ with TFP-mutE(NES). The upper panel shows the base line corrected instrumental response of successive additions of TFP-mutE(NES) (180 μM) to CRM1^HA^ (13 μM), at 20°C; the lower panel shows the integrated data (squares) and the fit of the binding isotherm by an independent binding sites model (solid line). χ^2^ /DoF (χ^2^ divided by the degree of freedom) of the fitting was 7.38 x 10^4^. The titration of negative control TFP-Cter (180 μM) onto CRM1^HA^ (14 μM) (circles) is also shown for comparison.

In order to validate the CRM1^HA^/NES binding relative affinities estimated by fluorescence anisotropy, and to further investigate the molecular details of these interactions, we have used ITC. Fig 5 shows the exothermic binding response of CRM1^HA^ titrated with TFP-mutE(NES). The thermodynamic parameters (Table 2) obtained after fitting the isotherm with a one binding site-model (Fig 5, lower panel) indicate that the interaction is enthalpically driven, while involving an entropic penalty. The recognition of NESs by CRM1 has been described to be mainly based on hydrophobic interactions, but also to involve several contacts of polar character [21–23], which could explain the favorable enthalpic term. In addition, desolvation of apolar groups of residues belonging to CRM1 and/or to the NES (e.g. Φ residues) would also favorably contribute to the enthalpic term. On the other hand, the entropic penalty could derive from the loss of conformational freedom of the NES motif, which is probably disordered in the free TFP construct and folds upon binding to CRM1. Of note, the thermodynamic signature of the CRM1^HA^/TFP-NES interaction is similar to that described for the CRM1/snurportin complex [21], even though this later complex involves a further CRM1 binding site in snurportin, in addition to the NES. Importantly, the CRM1^HA^/TFP-mutE(NES) affinity determined by ITC (K_D_ 1 μM) is higher than that of CRM1^HA^/TFP-mutA(NES) (2.4 μM), confirming the results derived from the fluorescence based binding assay.

**Table 2:** Thermodynamic parameters of CRM1^HA^ / NES interactions determined by ITC.

NES ligand	K_D_ (nM)	n	ΔH (kcal/mol)	-TΔS (kcal/mol)	ΔG (kcal/mol)
**YFP-PKI(NES)**	18 ± 3	1.01 ± 0.13	-13.7 ± 3.8	3.3 ± 3.7	-10.4 ± 0.1
**TFP-mutE(NES)**	1041 ± 41	0.80 ± 0.19	-15.4 ± 3.1	7.4 ± 3.1	-8.0 ± 0.0
**TFP-mutA(NES)**	2380 ± 698	0.54 ± 0.27	-14.5 ± 6.2	6.9 ± 6.1	-7.6 ± 0.1
**peptide ES**	76 ± 1	0.40 ± 0.05	-20.8 ± 1.1	11.3 ± 1.1	-9.6 ± 0.0
**peptide EL**	31 ± 1	0.51 ± 0.03	-19.8 ± 0.1	9.7 ± 0.1	-10.1 ± 0.0
**peptide AL**	57 ± 10	0.33 ± 0.06	-18.1 ± 0.9	8.4 ± 1.0	-9.7 ± 0.1
**peptide AS**	n.d.	n.d.	n.d.	n.d.	n.d.

As noted above, the K_D_ for the CRM1^HA^/YFP-PKI(NES) interaction we have estimated by fluorescence anisotropy (112 nM) was higher than the one previously reported in the literature (25 nM) [34]. When we analyzed the CRM1^HA^/YFP-PKI(NES) interaction by ITC, the K_D_ value obtained (18 nM, Table 2) perfectly agreed with previous reports. This finding suggests that we may have overestimated the K_D_ in the fluorescence anisotropy assay for this particular interaction due to the relatively high ligand concentration (50 nM) that we needed to use, as we were unable to obtain reliable fluorescence readouts at lower concentrations. It is important to note that this effect should not influence the estimation of the affinities for the rest of the CRM1^HA^/NES interactions, since their K_D_ values are well above 50 nM. In order to exclude a potential contribution of non-specific interactions (i.e. ascribed to the tag) to the binding affinities determined in the experiments with the TFP-NES constructs, we generated a negative control construct (TFP-Cter) that contains the last 9 C-terminal residues of wild type NPM. Titration of CRM1^HA^ with TFP-Cter produced small exothermic peaks (Fig 5), which may reflect a non-specific binding. Importantly, its low enthalpy makes unnecessary the correction of the CRM1^HA^ / NES binding phenomenon under study.

To further isolate the CRM1^HA^ / NES interaction from the putative influence of the tag protein (TFP, YFP), we have performed ITC binding experiments with untagged peptides corresponding to the NES motifs of mutant A and E. Moreover, to evaluate the contribution of the residue Φ^0^, which has been proposed to reinforce the recognition [23], we analyzed two versions of each NES. On one hand, the NES motifs as described by Bolli *et al*. [24], which correspond to the motifs tested as TFP-tagged constructs, and, on the other hand, a longer version of each motif including the residue Ile284, that could fulfill the role of Φ^0^. We designate these peptides AS, ES (for “short” version of the NES motifs of mutant A and E, respectively), and AL, EL (“long” versions). We titrated these peptides onto CRM1^HA^, observing binding responses for all except for AS, which showed no binding in our conditions. In line with the results of the TFP-NES peptides, the obtained parameters (Table 2) indicate that EL peptide binds CRM1^HA^ with slightly higher affinity than AL peptide (K_D_ 31 nM *vs*. 57 nM). Our data also provide insight into the role of the Φ^0^ residue in the interaction between the NES of mutant E and CRM1^HA^. Thus, the long version of the peptide displays higher affinity (K_D_ of 31 *vs*. 76 nM), suggesting that the presence of a Φ^0^ residue contributes to the binding strength. The ITC data also show that the peptides bind CRM1^HA^ with significantly higher affinity than the TFP constructs (K_D_ 76 *vs*. 1041 nM for the NES of mutant E), indicating that the TFP tag somehow hinders the interaction. The low binding stoichiometry values yielded, on the other hand, might result from a limited solubility of the peptides. Nevertheless, independently of the presence of the tag, both the compensation of an entropic penalty with favorable enthalpic change, as well as the comparison of relative affinities between different NESs, hold true, in agreement with the conclusions extracted with the TFP constructs. Although the difference in affinity for CRM1^HA^ between the NESs of mutants A and E is modest, the fact that the three experimental settings employed (anisotropy, ITC with TFP constructs and ITC with untagged peptides) reproducibly yield an affinity for the NES of mutant E approximately double that of mutant A, makes us consider it reliable. Altogether, therefore, the ITC results support the conclusions derived from fluorescence anisotropy measurements and strongly suggest that a hydrophobic residue (Ile284), upstream of the canonical NES, contributes to the binding energy.

### Thermodynamic characterization of the NPM/importin interaction

**Fig 6:**
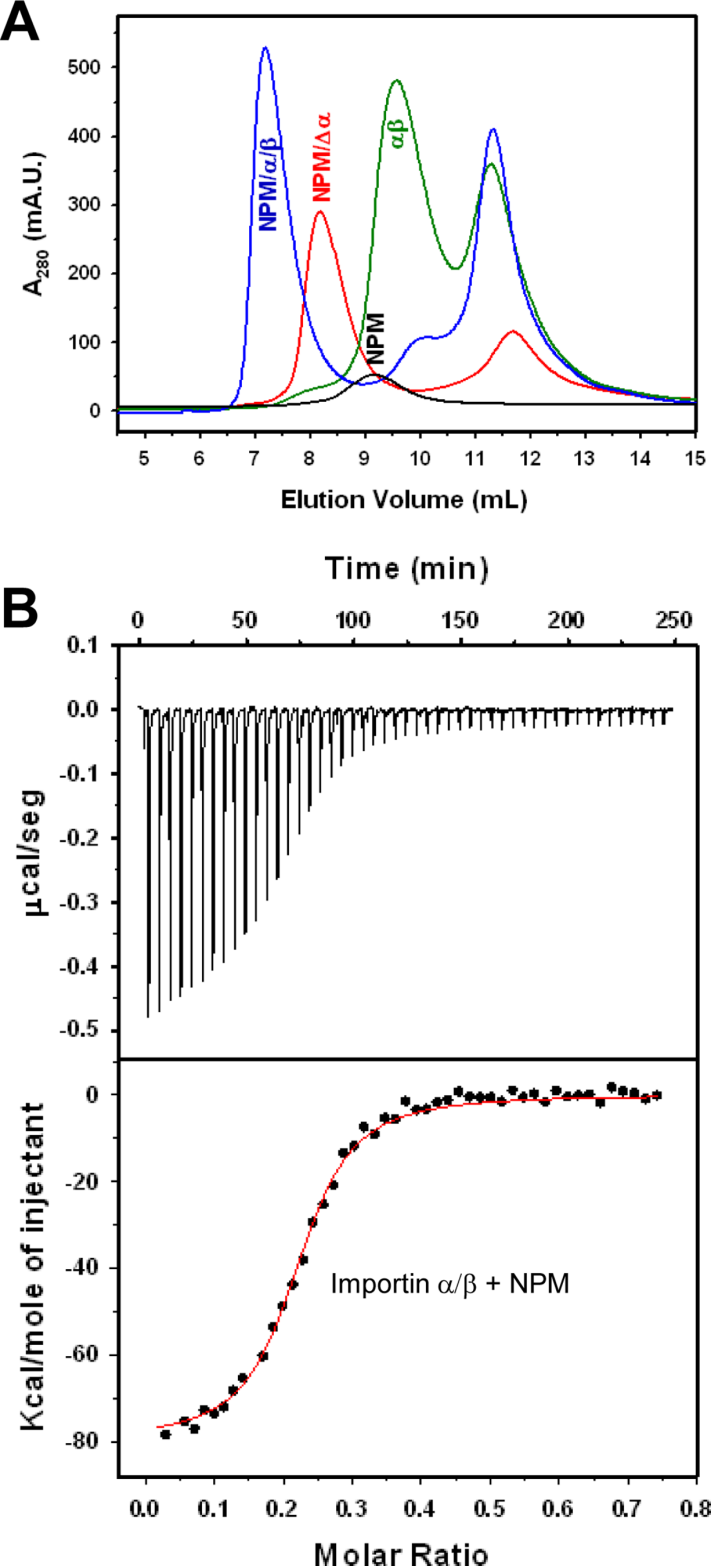
Analysis of importin α/β/NPM interaction by SEC and ITC. **(A)** Elution profiles of mixtures of 10 μM (pentamer) NPM with 70 μM importin Δα (red) or 70 μM importin α and 140 μM importin β (blue), and mixture of 70 μM importin α and 140 μM importin β (green). The control of 10 μM NPM (black) is also shown. The complexes are saturated, as evidenced by the absence of free NPM, and excess of free importins. Importin β was always added in molar excess to avoid the presence of free importin α, which also binds NPM, although with lower affinity. **(B)** Titration of importin α/β (21 μM) onto NPM (7.2 μM pentamer) (top panel) and fit of the binding isotherm by an independent binding sites model (bottom panel), with a χ^2^ / DoF of 3.22 x 10^6^.

In addition to CRM1-mediated nuclear export, other factors, including nuclear import and nucleolar retention contribute to determine NPM subcellular localization. We have previously characterized the binding of NPM to G-quadruplex forming DNA sequences, which may modulate its retention in the nucleolus [31]. Here, we aimed to thermodynamically characterize the recognition of NPM by the import machinery. To this end, we have analyzed the formation of complexes of full length, wild type, pentameric NPM with the importin α/β heterodimer, as well as with importin α alone and with a truncated form of importin α, lacking the autoinhibitory importin β-binding (IBB) domain (importin Δα). A ternary complex of NPM with the importin α/β heterodimer, as well as a complex with importin Δα, were readily detected by SEC (Fig 6A). Fig 6B shows the titration of importin α/β with NPM, and Table 3 summarizes the resulting thermodynamic parameters of NPM binding to the three types of ligands, using a fitting model of identical, independent binding sites. NPM bound to importin α/β and importin Δα with similar affinity, and to isolated full-length importin α with lower affinity.

**Table 3:** Thermodynamic parameters of the importin / NPM interactions determined by ITC.

NPM complex	K_D_ (nM)	n	ΔH (kcal/mol)	-TΔS (kcal/mol)	ΔG (kcal/mol)
**α/β-importin**	303 ± 92	5.0 ± 0.2	-16.7 ± 1.2	7.9 ± 1.2	-8.8 ± 0.2
**α-importin**	703 ± 347	5.0 ± 0.2	-10.9 ± 4.0	2.5 ± 3.7	-8.4 ± 0.3
**Δα-importin**	295 ± 20	4.9 ± 0.2	-14.3 ± 3.5	5.6 ± 0.3	-8.7 ± 0.1

Interestingly, our results indicate that the NPM pentamer can bind up to 5 importin α/β heterodimers (stoichiometry n = 5.0), indicating that the NLS motifs of all NPM subunits, located in their flexible linker regions, are accessible for simultaneous binding to importins. On the other hand, the thermodynamic signature of the binding phenomenon (Table 3) reveals an enthalpically driven interaction, related to polar contacts, whereas the entropic term contributes unfavorably. As discussed for CRM1/NES binding (see above) and for nucleoplasmin (NP) binding to importin [40], this unfavorable entropic term could be ascribed to the loss of conformational freedom of the NLS motif when it binds to the receptor. Our results indicate that importin α, which in the absence of importin β is inhibited through binding of its IBB domain to the NLS binding site, indeed recognizes NPM with lower affinity (Table 3), while the truncated mutant importin Δα mimics the affinity of the α/β heterodimer.

## Discussion

Aberrant CRM1-dependent, NES-mediated nucleocytoplasmic transport and cytoplasmic accumulation of AML-associated mutant forms of NPM (collectively termed NPMc+), has been attributed a driver role in the development of this malignancy [29, 30]. Interestingly, different AML mutants have been reported to greatly differ in the potency of their NESs, and it has been suggested that this can be balanced by a different retention in the nucleolus [24]. In the present study, we have quantitatively assessed the CRM1^HA^ binding affinity of the NESs mutationally acquired by two NPM mutants: mutant A (the most frequent mutant in AML) [27] and mutant E. We have found that, even though the NES of mutant E is indeed recognized by CRM1^HA^ with stronger affinity than that of mutant A, the difference (K_D_ 1 μM *vs*. 2.4 μM as determined by ITC with TFP-NES constructs) is not as pronounced as could be expected based on the results of previous cellular assays [24]. Thus, the observation that mutant A accumulates in the cytoplasm at similar or higher levels than mutant E when transfected in cells (ref. 24 and our own results) could be ascribed not only to a less efficient nucleolar retention but also to bearing a not so weak NES. We have also compared the affinities of mutant NPM NESs to that of the well-studied PKI NES. Our data indicate that the NES of mutant E is not as potent as that of PKI. These results suggest that *in vitro* binding assays, like the one used here, may be useful to discriminate between NESs considered to have the same “potency” in a cell-based assay [24]. Importantly, our thermodynamic characterization of CRM1^HA^ /NES interaction has allowed us to validate the estimations of relative affinities based on the fluorescence anisotropy assay, and has revealed further molecular details of the recognition. In particular, the analysis of the interaction between CRM1^HA^ and peptides corresponding to the NES motifs confirms, in a tag-free system, the conclusions derived from the experiments with TFP-NES constructs, and provides new insight into the role that the Φ^0^ residue might fulfill in the interaction. In some NESs, a hydrophobic residue upstream of the Φ^1^–Φ^4^ motif, termed Φ^0^, has been shown to increase the strength of binding to CRM1 [23]. In full length (mutant) NPM, Ile284 may fulfill the Φ^0^ role, and reinforce CRM1 recognition. In fact, formation of a tight complex between the Φ^0^–including NES of mutant A fused to GST and CRM1 / RanGTP has been described [25, 32]. However, the contribution of this potential Φ^0^ had not been yet directly assessed in a quantitative manner. We found that a longer version of the NES motif of mutant E including Ile284 binds CRM1^HA^ with higher affinity than a shorter motif lacking this residue (Table 2), supporting the view that Ile284 can be considered as a Φ^0^ residue in mutant NPM NESs.

Our study has explored the molecular determinants of both NPM export (CRM1/NES) and import (importin α/β/NLS). The affinity of NPM for importin α/β (K_D_ 303 nM) is lower than that of the homologous protein nucleoplasmin (K_D_ 57 nM) [40]. This lower affinity could be related to the fact that NPM NLS includes one basic residue less in the major cluster of the bipartite motif than NP NLS. The importin α/β/NPM binding parameters we report suggest that NPM import is nevertheless efficient. This, together with the very low affinity of CRM1 for wild type NPM and significant avidity for nucleolar components, explains the almost exclusively nuclear (nucleolar) localization of wild type NPM. In the case of AML-associated NPM mutants, we can assume that the mutations, in a distant region of the protein, do not affect the importin/NLS interaction. Instead, regarding their export efficiency, one must consider that our estimation of NES/CRM1 affinities is based on the use of a CRM1 mutant and isolated NESs. The CRM1 recognition of the NES motifs must be significantly affected by the protein context. We have attempted to characterize the CRM1 binding properties of full length NPM mutants and have shown, for the first time, the formation of complexes between CRM1^HA^ and NPMmutA or NPMmutE (Fig 3B). The susceptibility of their unfolded C-terminal domain to proteolytic degradation hampers, however, the attainment of quantitative information. While the binding parameters of full length AML mutants for CRM1 cannot be directly assessed because of these experimental caveats, their affinities could indeed differ from those of isolated NES motifs: On one hand, the additional NESs (i.e. acquired and intrinsic) in the protein (note that it is pentameric) may contribute to the binding. Furthermore, even if the intrinsic potency of the NES of mutant E is slightly higher than that of mutant A, it should be considered that the more complete unfolding of mutant A [11], would favor its recognition by CRM1, and therefore its export, in the full length protein.

While it is clear that the acquired NESs of AML mutants are critical to displace NPMc+ to the cytoplasm, it seems that NPM is normally recognized by CRM1 very weakly, resulting in a mostly nucleolar localization of the wild type protein, at least during interphase of the cell cycle [43]. However, the mechanistic details of physiological nuclear export of wild type NPM remain elusive, and the identity of the critical NES motif(s) in the wild type protein is still not clear, since all of the tested candidates have shown low or no activity when assayed in isolation [24, 25, this study]. It remains possible that several NES motifs might cooperate in directing NPM export; in fact, it has been reported that both the intrinsic 94–102 amino acid sequence and the acquired NES motif are necessary for efficient export of mutant A [33]. Besides, the fact that NPM is oligomeric and displays several NES motifs might promote CRM1 binding due to multipartite recognition of individually weak NES epitopes, as discussed elsewhere [21]. On the other hand, NPM export might be induced under certain conditions, or modulated by post-translational modifications. In particular, phosphorylation of certain residues (such as threonine 95 and serine 125 in the core domain) has been suggested to promote opening of the otherwise compact core [26], and this could affect exposure of the putative NESs in that domain. Furthermore, export might be facilitated by release of NPM from nucleolus, for instance upon DNA damage [7]. In this context, our results suggest that treatment with DNA-damaging agents may promote NPM export in cells.

Apart from AML, other types of leukemia have been related to altered nucleocytoplasmic transport of proteins and/or alterations of the nuclear export machinery itself [44]. Not only NPM and CRM1 are targets in leukemia, but their interaction can be considered a therapeutic target [32], since NPM aberrant export concerns 30% of all AML cases [28]. In particular, inhibiting NPM/CRM1 binding, perhaps with peptides mimicking the NES of AML mutants might represent an interesting strategy. It is clear that any progress on the role of NPM and CRM1 in disease will require a detailed knowledge of their interaction. We have explored the molecular basis of NPM recognition by CRM1 and by importin α/β. Our results indicate that, whereas wild type NPM is inefficiently exported, AML-associated mutations dramatically enhance its ability to bind CRM1. Based on our quantitative binding data, minor differences in affinity between the NES motifs of different AML mutants can be correlated with their aberrant export by CRM1.

## Supporting Information

S1 Fig A novel candidate NES (cNES) in NPM is not active in a nuclear export assay. **(A)** Aminoacid sequence of a novel candidate NES (cNES) predicted in NPM using the bioinformatic tool Wregex [42]. The “relaxed” configuration in the Wregex Search page was applied. The hydrophobic residues (Φ^1^-Φ^4^) that conform to the NES consensus (shown below) are highlighted in red. **(B, C).** Results of an *in vivo* nuclear export assay to test the activity of NPM cNES. A cDNA encoding the NPM sequence ^264^VEAKFINYVKNCFRMTDQE was cloned into the Rev(1.4)-GFP plasmid [38]. The resulting plasmid Rev(1.4)-NPMcNES-GFP was transfected into HeLa cells, and the export assay was carried out as described [38], using actinomycin D (Act D) to block Rev(1.4)-mediated nuclear import. As a negative control (c-), the empty Rev(1.4)-GFP plasmid was used. As a positive control (c+) a Rev(1.4)-GFP-derived plasmid containing a functional NES (ELM3) previously characterized [García-Santisteban I, Bañuelos S, Rodríguez J.A. (2012) Biochem J: 441: 209–217] was included. Fluorescence microscopy images in panel B show the localization of the different proteins. DAPI was used to counterstain the nuclei. Graphs in panel C indicate the percentage of cells showing nuclear (N), nucleo/cytoplasmic (N/C) or cytoplasmic (C) localization of each fluorescent protein. More than 200 cells were counted per sample. These results indicate that the predicted NPM cNES is not functional as a nuclear export signal.
(TIF)

S2 Fig Cell localization of wild type NPM and AML-associated mutants. HeLa cells were transfected with YFP-NPM (top), YFP-NPMmutE (middle) or YFP-NPMmutA (bottom panel). Cells were fixed 24 h after transfection, and localization of NPM evaluated analyzing YFP (left) and Hoechst 33258 fluorescence to counterstain nuclei (right panels).
(TIF)

S3 Fig The analysis of mutant NPM preparations by mass spectrometry (MS) indicates significant proteolytic degradation. The proteins, at approximately 30 mg / mL in 25 mM Tris/HCl, pH 7.5, 500 mM NaCl, 1 mM DTT, 10% glycerol, were diluted 50 times in 50% acetonitrile, 0.2% formic acid. The samples were directly injected into a Q-Tof Micro (Waters) mass spectrometer and MS spectra were manually acquired in the m/z range 500–1700. Protein intact mass was determined by MaxEnt1 software (Waters) and default deconvolution parameters were used. Mass ranges were selected based on the protein sequence and the software was set to iterate to convergence. MS results are shown for wild type NPM, NPMmutA and NPMmutE. Processed spectra in the range 20000–35000 Da are shown (left) and the masses of the major species detected are listed (right), and compared with the theoretical masses of the full length proteins (which include residues GS at the N-terminus for cloning reasons), as well as the most plausible assignment to C-terminally truncated species (right). Whereas the mass of wild type NPM agrees with the expected one, both mutants A and E display, apart from the full length species, a number of degraded products. Although this type of MS analysis does not allow to reliably comparing the relative quantities of the different molecules, it suggests that a) full length mutants are minority and b) mutant E is more degraded than mutant A. It is important to note that all the truncated forms lack the NES motif.
(TIF)

S4 Fig TFP-NES / CRM1^HA^ complex formation shown by Ni-NTA chromatography. 2.5 mg of the His-tagged TFP construct were loaded on a 1 mL Histrap FF (GE Healthcare) in buffer 50 mM Tris/HCl, pH 8.0, 500 mM NaCl, 1 mM TCEP, 2 mM MgCl_2_, 5% glycerol, containing 15 mM imidazole. After washing with the same buffer, 1 mL of CRM1^HA^ at 18 μM was injected at 0.1 mL/min flow rate. Unbound CRM1^HA^ was collected, and then bound proteins were eluted with 2 M imidazole. SDS-PAGE of elution fractions: Unbound (free) CRM1^HA^ and the fraction eluted with imidazole, containing both the His-tagged TFP construct along with bound CRM1^HA^.
(TIF)
