# Supplementary figures and images for: Leukemia-Associated Mutations in Nucleophosmin Alter Recognition by CRM1: Molecular Basis of Aberrant Transport
Source: PLoS One. 2015 Jun 19;10(6):e0130610. doi: 10.1371/journal.pone.0130610 (PMC4474691; doi:10.1371/journal.pone.0130610)

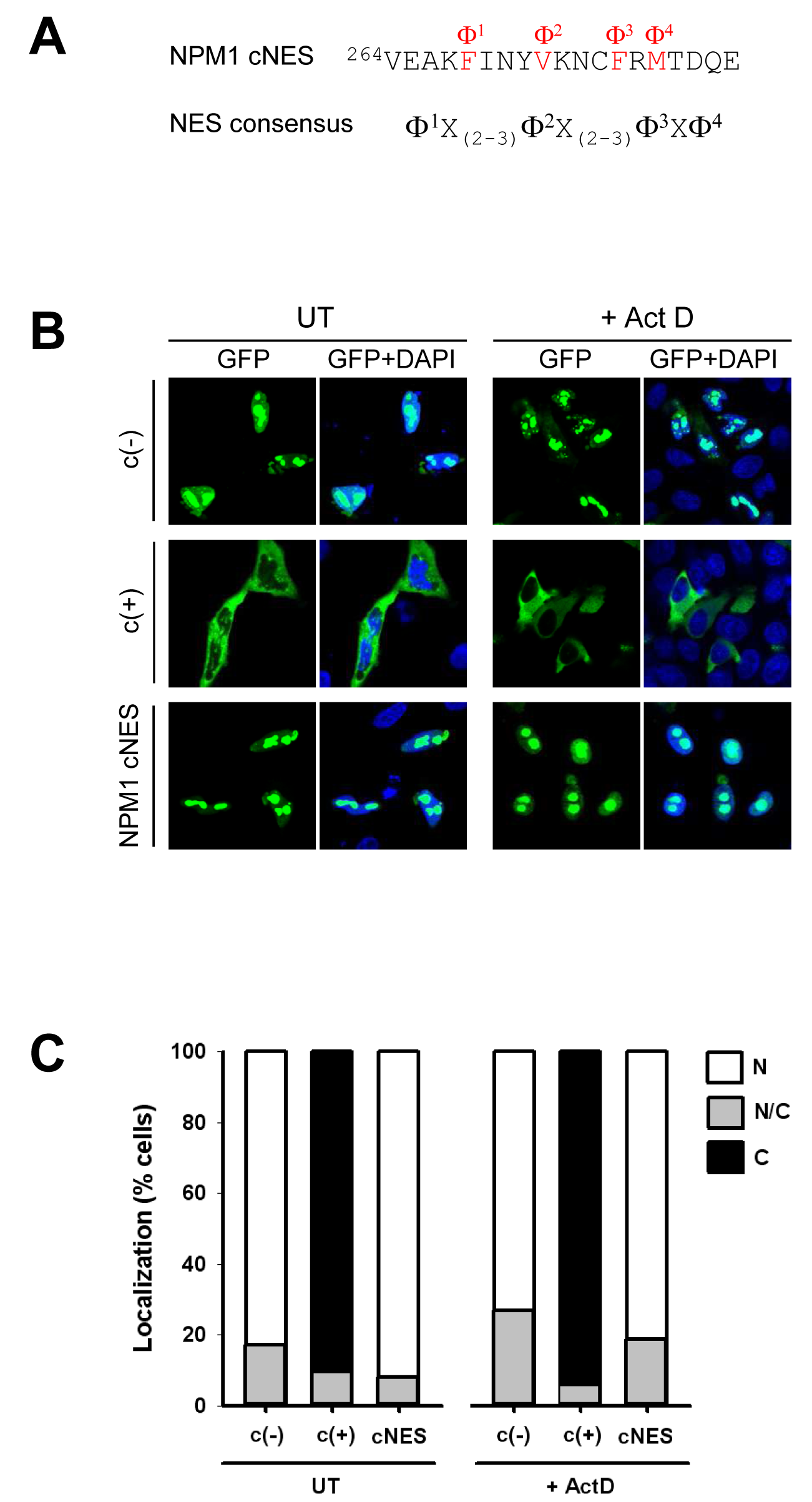

Supplement: S1 Fig — (A) Aminoacid sequence of a novel candidate NES (cNES) predicted in NPM using the bioinformatic tool Wregex [42]. The “relaxed” configuration in the Wregex Search page was applied. The hydrophobic residues (Φ1-Φ4) that conform to the NES consensus (shown below) are highlighted in red. (B, C). Results of an in vivo nuclear export assay to test the activity of NPM cNES. A cDNA encoding the NPM sequence 264VEAKFINYVKNCFRMTDQE was cloned into the Rev(1.4)-GFP plasmid [38]. The resulting plasmid Rev(1.4)-NPMcNES-GFP was transfected into HeLa cells, and the export assay was carried out as described [38], using actinomycin D (Act D) to block Rev(1.4)-mediated nuclear import. As a negative control (c-), the empty Rev(1.4)-GFP plasmid was used. As a positive control (c+) a Rev(1.4)-GFP-derived plasmid containing a functional NES (ELM3) previously characterized [García-Santisteban I, Bañuelos S, Rodríguez J.A. (2012) Biochem J: 441: 209–217] was included. Fluorescence microscopy images in panel B show the localization of the different proteins. DAPI was used to counterstain the nuclei. Graphs in panel C indicate the percentage of cells showing nuclear (N), nucleo/cytoplasmic (N/C) or cytoplasmic (C) localization of each fluorescent protein. More than 200 cells were counted per sample. These results indicate that the predicted NPM cNES is not functional as a nuclear export signal. (TIF) [file pone.0130610.s001.tif]

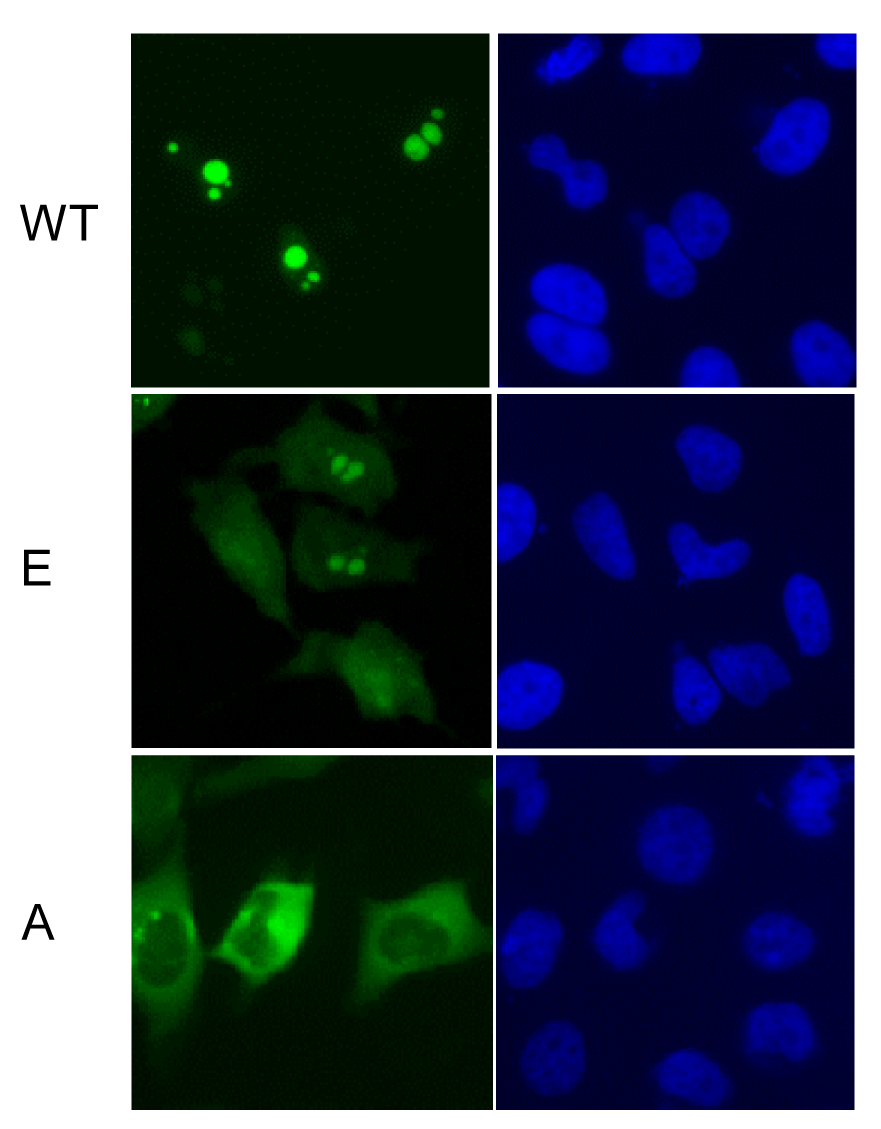

Supplement: S2 Fig — HeLa cells were transfected with YFP-NPM (top), YFP-NPMmutE (middle) or YFP-NPMmutA (bottom panel). Cells were fixed 24 h after transfection, and localization of NPM evaluated analyzing YFP (left) and Hoechst 33258 fluorescence to counterstain nuclei (right panels). (TIF) [file pone.0130610.s002.tif]

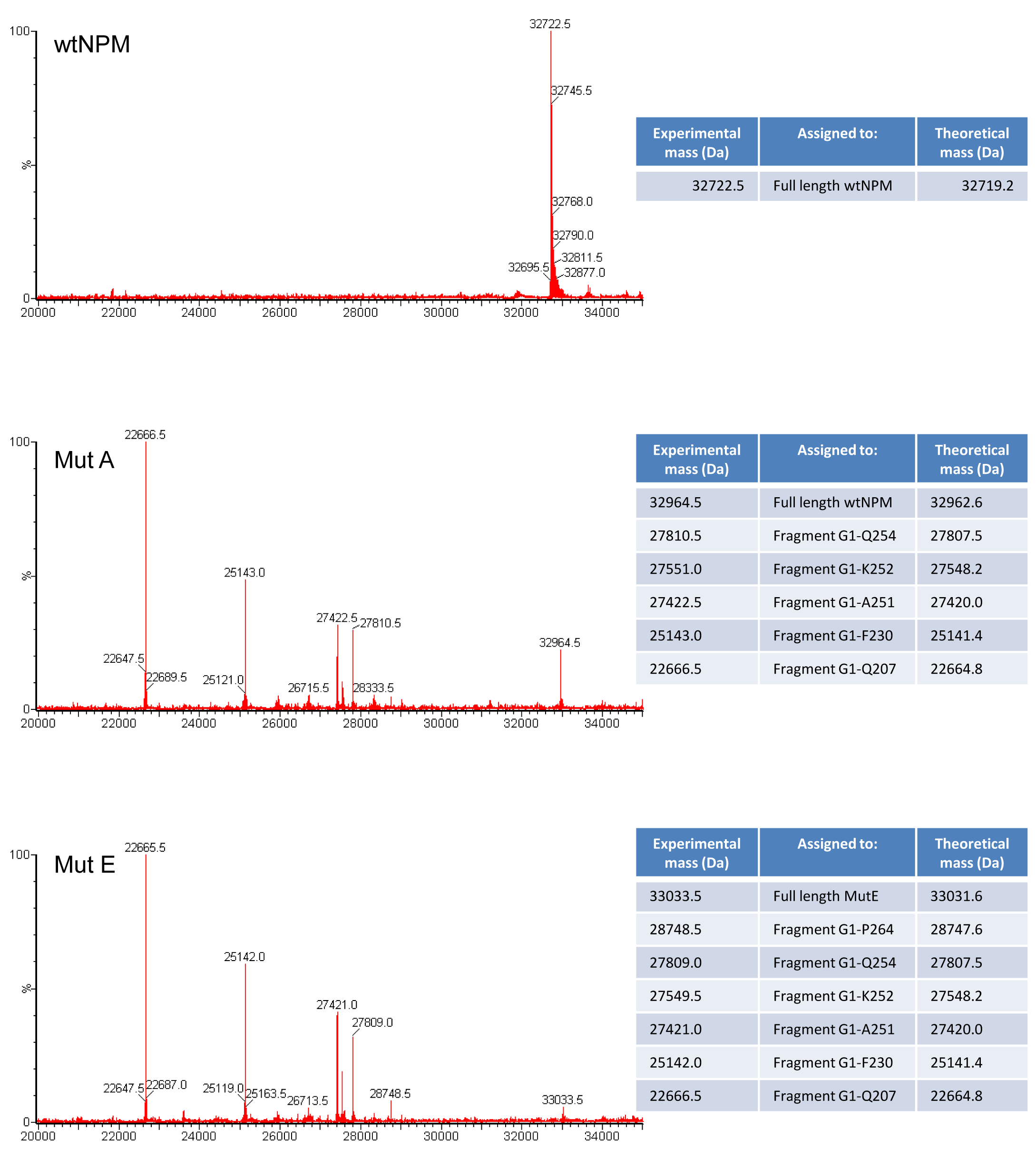

Supplement: S3 Fig — The proteins, at approximately 30 mg / mL in 25 mM Tris/HCl, pH 7.5, 500 mM NaCl, 1 mM DTT, 10% glycerol, were diluted 50 times in 50% acetonitrile, 0.2% formic acid. The samples were directly injected into a Q-Tof Micro (Waters) mass spectrometer and MS spectra were manually acquired in the m/z range 500–1700. Protein intact mass was determined by MaxEnt1 software (Waters) and default deconvolution parameters were used. Mass ranges were selected based on the protein sequence and the software was set to iterate to convergence. MS results are shown for wild type NPM, NPMmutA and NPMmutE. Processed spectra in the range 20000–35000 Da are shown (left) and the masses of the major species detected are listed (right), and compared with the theoretical masses of the full length proteins (which include residues GS at the N-terminus for cloning reasons), as well as the most plausible assignment to C-terminally truncated species (right). Whereas the mass of wild type NPM agrees with the expected one, both mutants A and E display, apart from the full length species, a number of degraded products. Although this type of MS analysis does not allow to reliably comparing the relative quantities of the different molecules, it suggests that a) full length mutants are minority and b) mutant E is more degraded than mutant A. It is important to note that all the truncated forms lack the NES motif. (TIF) [file pone.0130610.s003.tif]

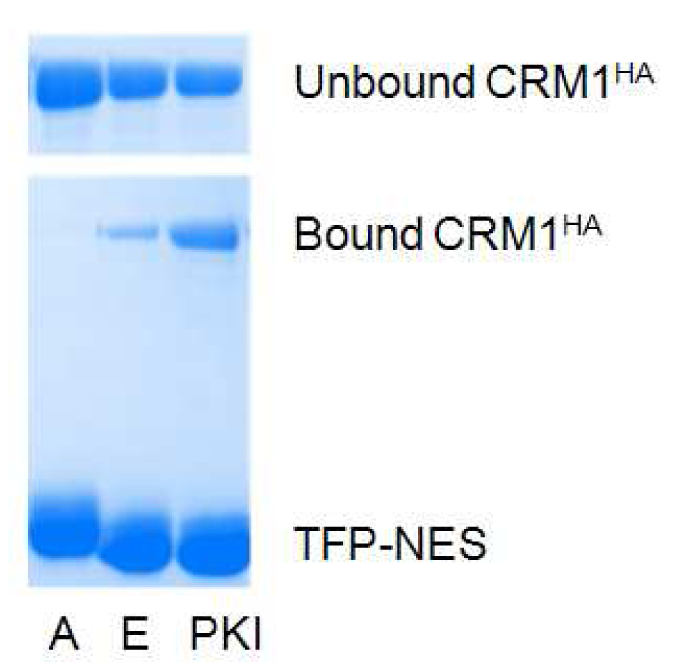

Supplement: S4 Fig — 2.5 mg of the His-tagged TFP construct were loaded on a 1 mL Histrap FF (GE Healthcare) in buffer 50 mM Tris/HCl, pH 8.0, 500 mM NaCl, 1 mM TCEP, 2 mM MgCl2, 5% glycerol, containing 15 mM imidazole. After washing with the same buffer, 1 mL of CRM1HA at 18 μM was injected at 0.1 mL/min flow rate. Unbound CRM1HA was collected, and then bound proteins were eluted with 2 M imidazole. SDS-PAGE of elution fractions: Unbound (free) CRM1HA and the fraction eluted with imidazole, containing both the His-tagged TFP construct along with bound CRM1HA. (TIF) [file pone.0130610.s004.tif]
